# Perceived barriers in the use of ultrasound in developing countries

**DOI:** 10.1186/s13089-015-0028-2

**Published:** 2015-06-19

**Authors:** Sachita Shah, Blaise A. Bellows, Adeyinka A. Adedipe, Jodie E. Totten, Brandon H. Backlund, Dana Sajed

**Affiliations:** Division of Emergency Medicine, University of Washington, Box 359702, 1CT89, 325 9th, Ave, Seattle, WA 98104-2499 USA; Department of Emergency Medicine, Massachusetts General Hospital, Zero Emerson Place, Suite 3B, Boston, MA 02114 USA

**Keywords:** Ultrasound, Global health, Public health

## Abstract

**Background:**

Access to ultrasound has increased significantly in resource-limited settings, including the developing world; however, there remains a lack of sonography education and ultrasound-trained physician support in developing countries. To further investigate this potential knowledge gap, our primary objective was to assess perceived barriers to ultrasound use in resource-limited settings by surveying care providers who practice in low- and middle-income settings.

**Methods:**

A 25-question online survey was made available to health care providers who work with an ultrasound machine in low- and middle-income countries (LMICs), including doctors, nurses, technicians, and clinical officers. This was a convenience sample obtained from list-serves of ultrasound and radiologic societies. The survey was analyzed, and descriptive results were obtained.

**Results:**

One hundred and thirty-eight respondents representing 44 LMICs including countries from the continents of Africa, South America, and Asia completed the survey, with a response rate of 9.6 %. Ninety-one percent of the respondents were doctors, and 9 % were nurses or other providers. Applications for ultrasound were diverse, including obstetrics (75 %), DVT evaluation (51 %), abscess evaluation (54 %), cardiac evaluation (64 %), inferior vena cava (IVC) assessment (49 %), Focused Assessment Sonography for Trauma (FAST) exam (64 %), biliary tree assessment (54 %), and other applications. The respondents identified the following barriers to use of ultrasound: lack of training (60 %), lack of equipment (45 %), ultrasound machine malfunction (37 %), and lack of ultrasound maintenance capability (47 %). Seventy-four percent of the respondents wished to have further training in ultrasound, and 82 % were open to receiving distance learning or telesonography training. Subjects used communication tools including Skype, Dropbox, emailed photos, and picture archiving and communication system (PACS) as ways to communicate and receive feedback on ultrasound images.

**Conclusions:**

Health care providers in the developing world identify lack of training as a primary barrier to regular use of ultrasound in their practice. While equipment requirements including maintenance and cost of machines are also important factors, future research is warranted on best practices for training methods, including telesonography and distance learning to enhance ultrasound use in low-resource settings.

## Background

The use of ultrasound in the developing world has increased exponentially over the past decade and encompasses a diverse range of applications [1–4]. As ultrasound machines have become smaller, more reliable, and less expensive, their availability in developing countries has increased dramatically [5]. Though there is significant literature regarding the feasibility, utility, and applications for which ultrasound may be used, there is a paucity of evidence regarding optimal training and barriers to using ultrasound in low-resource settings. One review on the topic by LeGrone et al. suggests that the majority of providers using ultrasound in developing countries have received less training than what is required to meet the minimum standards set forth by the World Health Organization (WHO) [6]. These standards recommend a minimum of 3–6 months of directed education including 300–500 ultrasound examinations [7]. Major barriers to completing this training included lack of trained teachers and inability to spend enough time at training sites far from a provider’s primary clinical site. However, this study was limited in that, as a literature review, it only examined ultrasound training which had been previously described in published articles [6]. Our goal was to identify perceived barriers to the use of ultrasound in developing countries, to identify if this included inadequate training, and to explore if providers would be interested in using distance learning modules to further their training in the use of ultrasound.

## Methods

The study was approved by the University of Washington Institutional Review Board. A 25-question online survey (Appendix) was distributed by email to a convenience sample of 1435 practitioners with potential experience using ultrasound in low-and middle-income countries (LMICs) and who were members of ultrasound interest groups including the Emergency Ultrasound Section of the American College of Emergency Physicians, the Academy of Emergency Ultrasound Section of the Society for Academic Emergency Medicine, and the American Institute of Ultrasound in Medicine Global Health Interest Group. These providers include members of Partners in Health, Doctors Without Boarders, and International Medical Care and Global Emergency Care Collaborative who are known to work in LMICs with ultrasound and to be part of these list-serves. The survey was also directly emailed to some providers of the above organizations who are known to work with ultrasound in LMICs. These providers included doctors, nurses, technicians, and clinical officers. Consent was obtained via the first question in the survey. The survey contained demographic questions including level of training, clinical facility, and country of clinical work. The survey contained ten “yes/no” questions regarding surgical capability at the clinical site, radiographic capability at the site, ultrasound availability, and history of formal and informal ultrasonography training. The survey contained 15 multiple-answer questions regarding the types of ultrasound studies participants perform, types of probes available for ultrasound machines, technical problems encountered with ultrasound machines, availability and type of repair for ultrasound machines, problems with image interpretation, and the support of radiology staff for image interpretation. The survey was analyzed with simple statistics, and descriptive results were obtained from multiple-answer and free text answer choices.

## Results

One hundred and thirty-eight providers completed the survey giving a response rate of 9.6 %. Respondents were from 44 countries covering Africa, South America, and Asia (Table 1). Ninety-one percent of the respondents were doctors, and 9 % were nurses or other providers. Applications for ultrasound were diverse, including obstetrics (75 %), DVT evaluation (51 %), abscess evaluation (54 %), cardiac evaluation (64 %), inferior vena cava (IVC) assessment (49 %), Focused Assessment Sonography for Trauma (FAST) exam (64 %), biliary tree assessment (54 %), and other applications. The respondents identified the following barriers to use of ultrasound (Table 2): lack of training (60 %), lack of equipment (45 %), ultrasound machine malfunction (37 %), and lack of maintenance (47 %). Seventy-four percent of the respondents wished to have further training in ultrasound, and 82 % were open to receiving distance learning or telesonography training. Subjects used communication tools including Skype, Dropbox, emailed photos, and picture archiving and communication system (PACS) as ways to communicate and receive feedback on ultrasound images.

**Table 1:** Countries represented

Country	Number of respondents working in that country
Haiti	21
Rwanda	15
India	10
Uganda	8
Malawi	7
Tanzania	7
Mexico	6
USA	6
Liberia	5
Burundi	5
Ghana	4
Kenya	4
Nepal	4
Somaliland	3
Bangladesh	2
Ethiopia	2
Guatemala	2
Mali	2
Mongolia	2
Nigeria	2
Pakistan	2
Peru	2
Saudi Arabia	2
Sierra Leone	2
South Sudan	2
Turkey	2
Australia	1
Brazil	1
Borneo	1
Cameroon	1
Ecuador	1
Gabon	1
Guinea	1
Honduras	1
Indonesia	1
Italy	1
Laos	1
Lebanon	1
Lesotho	1
Monrovia	1
Somalia	1
Syria	1
Tibet	1

**Table 2:** Barriers to using ultrasound

	Percentage of respondents who have experienced this barrier to ultrasound use
Lack of training	59.9
Cost of maintaining/obtaining/updating machines	50.0
Lack of reliable maintenance to fix machine	47.0
Lack of equipment	45.5
Lack of internet to tele-communicate for support	43.9
No support personnel to answer questions	38.6
Machine breaking	37.1
Lack of gel	32.6
Trained personnel in ultrasound leaving the site	31.8
Lack of electricity or power	31.0
Lack of support of point of care ultrasound from the hospital administration	25.0
Discomfort in image interpretation	25.0
Lack of support of point of care ultrasound from the radiology department	23.5
Discomfort in using ultrasound to make images	15.2
Too much time to get ultrasound and perform exam	14.4
Liability concerns	9.1
Adequate coverage by radiology—eliminating need to perform own exam	3.8

## Discussion

Ultrasound use has been increasing at a rapid rate in the developing world due to improved portability, durability of machines, and the recognition of the diverse diagnostic capabilities that ultrasound can offer in austere settings [5]. While there has been significant ultrasound implementation in the developing world, much of this has been equipment only without training, in settings where formal schooling for sonography and radiology specialty training for physicians does not exist, leaving care providers to scramble for knowledge of clinician-performed point-of-care ultrasound applications. Review of the literature reveals much of the ultrasound training that is currently documented in developing countries may be short-term training under the auspice of foreign non-governmental organizations (NGOs) and aid projects that may not provide long-term repeated trainings, machine maintenance, or help with image interpretation after the initial training period.

Providers in the developing world continue to encounter significant barriers to the use of ultrasound, including lack of training, machine malfunction, and inability to perform maintenance on existing machines. Many providers are receptive to the idea of distance learning modules, which will be an important area of future research and implementation. Further research should be directed towards the optimal type and duration of training for each specific point-of-care ultrasound application, such as the FAST exam and limited obstetric ultrasound, and whether training needs differ for different learner types (e.g., medical doctors versus clinical officers or nurses), in order to develop distance-learning modules which are concise and content rich. Further investigations into an internationally recognized standard of training or certificate program would also be of benefit to help ensure quality of training and optimal application of ultrasound technology [8]. Similarly, developing a standardized monitoring-and-evaluation protocol for providers in the developing world to have an objective measure of the quality of their exams, image acquisition, and image interpretation would be of tremendous benefit for ongoing quality of care. Future research efforts should also focus on the sustainability of ultrasound use, including the feasibility of “train the trainer” approaches to increasing local provider investment in continuing ultrasound programs at their home clinical sites.

The limitations of this study include low response rate, the use of a yet un-validated survey, the use of a convenience sample from US-based ultrasound list-serves, and possible recall bias as providers were asked to self-report on past experiences. These limitations are at least in part because there is no single, dedicated list-serve for LMIC ultrasound practitioners, and it is unlikely that the majority of subscribers to the list-serves sampled practice in LMICs. We acknowledge that the majority of providers in LMICs are non-physician clinicians, which are not represented well in this survey which may also lead to a lack of generalizability. We hope to improve on these limitations by modifying this pilot survey and distributing it to the nurse in charge at 50 facilities within Uganda and Zambia. We anticipate a high response rate and more generalizable results from this focused demographic.

## Conclusions

Ultrasound has the capability to markedly improve diagnostic capabilities in the developing world; however, the success of this diagnostic modality is operator as well as equipment dependent. Our study identifies current barriers to ultrasound use in low-resource settings including lack of ultrasound training and sufficient equipment, as well as lack of access to reliable machine maintenance. Further research should be directed towards developing concise, content-rich distance learning modules, programs for creating sustainable training opportunities, and a recognized international standard of assessment and certification in ultrasound.

## Appendix

### Survey

Ultrasound Survey to assess Training Needs and Perceived Barriers to Point of Care Ultrasound in the Developing World

You should complete the survey if you currently practice in a developing country, OR if you have practiced in a developing country, please fill the survey out based on your work in that country (eg. You are American but taught in Nicaragua, so fill out the answers as if you are still in the hospital in Nicaragua- what are the needs and barriers?)

Question 1. All questions in this survey pertain to your practice in the developing world and not your practice in the United States or a developed country. (I accept)

Question 2. Optional Contact Information. Write in: Name, Email Address, and Hospital Name/Location of the Developing Country

Question 3. What is your level of training? (Doctor/Nurse/Other)

Question 4. In the developing country, what type of facility do you work in? Check all that apply. (Hospital/Clinic/Rural/Urban-Countryside, more the 1 hour drive from a city/Other_)

Question 5. What developing country do you work in? (Write In)

Question 6. Please respond Yes or No to the following questions. (Please respond to the questions about your practice in the developing world.)

- Do you have surgical capacity at your site/an operating room? (Yes/No)
- Do you have an X-ray machine at your site? (Yes/No)
- Do you have access to ultrasound in your clinical practice? (Yes/No)
- Do you have training in ultrasound? (Yes/No)
- Have you had informal ultrasound teaching from a colleague (not part of a formal course)? (Yes/No)

Question 7. Have you taken a formal (with lectures) ultrasound course? If yes, please write in the length and name of the course. (Yes/No/If yes, please include length and name:)

Question 8. If you have had no prior training, are you self taught? (Yes/No/Not Applicable)

Question 9. How often do you use the ultrasound? Check which applies. (On a daily basis/On a weekly basis/On a monthly basis/Rarely)

Question 10. What sort of applications do you use ultrasound for? (Check all that apply) (OB-GYN/Ocular/Deep Venous Thrombosis/Skin and Soft Tissue (Abscess or Cellulitis)/Fractures/Nerve Blocks/Evaluation of retropharyngeal abscesses/Cardiac Evaluation/Inferior Vena Cava For Volume Assessment/ FAST for trauma/Pneumothorax/Evaluation of the biliary tree-gall bladder/Evaluation of the kidneys/Ultrasound guided procedures: Central Venous Lines/ Ultrasound guided procedures: Pericardiocentesis/Ultrasound guided procedures: Paracentesis/Ultrasound guided procedures: Thoracentesis/Ultrasound guided procedures: Abscess evaluation and drainage/Ultrasound guided procedures: Difficult Peripheral IVs/Biopsy Guidance/Liver/Spleen/Thyroid/Lymphadenopathy/Ascites/Other)

ULTRASOUND EQUIPMENT QUESTIONS

Question 11. How many (please check one) and what kind of machines do you have access to (write in machine)? (0-10)

Question 12. What probes do you have for your machines? (Linear (superficial)/Cardiac (phased array)/Curved abdominal (OB probe)/Endocavitary(vaginal probe)/I don't know)

BARRIERS

Question 13. Has the ultrasound broken to your knowledge since it was first used at your site? (Yes/No)

Question 14. If yes, what has broken? (Cord, power supply/Probes/Motherboard-Main Machine/Other)

Question 15. If your machine has broken, did you repair it: (Locally/Within country/Send it abroad/ Has not broken/Did not repair)

Question 16. Check all that apply: These are the barriers I have encountered in using ultrasound. (Lack of training/Lack of needed equipment like machines or probes/Lack of gel/Lack of electricity or power/Lack of internet ability to tele-communicate for support/No support personnel available to answer questions/Trained personnel in ultrasound leaving the site/Machine breaking/Lack of support of point of care ultrasound from radiology department/Lack of support of point of care ultrasound from hospital administration/Lack of reliable maintenance to fix machine/Cost of maintaining/obtaining/updating machines/Discomfort using ultrasound to make images/Discomfort in image interpretation/Liability concerns/Adequate coverage by radiology- eliminating need to perform own exam/Too much time to get ultrasound machine and perform exam)

Question 17. Please describe additional barriers to using ultrasound that are not listed above.

Question 18. Has lack of medical professionals willing to learn ultrasound been a problem at your site? (Yes/No)

Question 19. Do you have help to interpret your ultrasound images by a colleague or radiologist? (Yes/No)

Question 20. If you send out a question to get help with ultrasound interpretation how long until you get a response: Select one. (1 day/under 1 week/under 1 month/more than a month)

Question 21. If you discover a finding on ultrasound that would mean a patient needs surgery or care not available at your site, do you have the ability to transfer the patient to a capable facility? (Yes/No)

Question 22. (Check all that apply) In your practice, do you have access to: (Reliable Internet/Reliable power supply/Skype/PACS or other wireless upload of images from machine to web)

Question 23. In your practice, have you ever sent ultrasound images to someone for review? (Yes/No)

Question 25: What resources for learning… (Yes/No)

Question 24. If yes, what method did you use: (Check all that apply) (Cell phone picture/Emailed photo or video/Skype real time/PACS or other wireless/transmission/Other: _).

Question 25. What resources for learning ultrasound (books, courses, websites) have you used and found helpful: (PIH manual/ www.sonoguide.com/ Other:_)

Question 26. Please respond to the following questions with your level of agreement.

- I would like more ultrasound training. (1- Strongly Disagree/2- Moderately Disagree/3- Slightly Disagree/4- Slightly Agree/5- Moderately Agree/6- Strongly Agree)
- I would like to participate in a web-based, virtual ultrasound course for point of care ultrasound skills. (1- Strongly Disagree/2- Moderately Disagree/3- Slightly Disagree/4- Slightly Agree/5- Moderately Agree/6- Strongly Agree)

## References

[1] Adler D, Mgalula K, Price D, Taylor O (2008). Introduction of a portable ultrasound unit into the health services of the Lugufu refugee camp, Kigoma District, Tanzania. Int J Emerg.

[2] Dean AJ, Ku BS, Zeserson EM (2007). The utility of handheld ultrasound in an austere medical setting in Guatemala after a natural disaster. Am J Disaster Medicine.

[3] Fagenholz PJ, Gutman JA, Murray AF, Noble VE, Thomas SH, Harris NS (2007). Chest ultrasonography for the diagnosis and monitoring of high altitude pulmonary edema. Chest.

[4] Kimberly HH, Murray A, Mennicke M, Liteplo A, Lew J, Bohan JS, Tyer-Viola L, Ahn R, Burke T, Noble VE (2010). Focused maternal ultrasound by midwives in rural Zambia. Ultrasound Med Biol.

[5] Sippel S, Muruganandan K, Levine A, Shah S (2011). Review article: use of ultrasound in the developing world. Int J Emerg Med.

[6] LaGrone LN, Sadasivam V, Kushner AL, Groen RS (2012). A review of training opportunities for ultrasonography in low and middle income countries. Trop Med Int Health.

[7] World Health Organization (1998). Training in diagnostic ultrasound: essentials, principles and standards.

[8] Shah SP, Epino H, Bukhman G, Umulisa I, Dushimiyimana JM, Reichman A, Noble VE (2009). Impact of the introduction of ultrasound services in a limited resource setting: rural Rwanda 2008. BMC Int Health Hum Rights.

